# Nanofluid flow with autocatalytic chemical reaction over a curved surface with nonlinear thermal radiation and slip condition

**DOI:** 10.1038/s41598-020-73142-9

**Published:** 2020-10-27

**Authors:** Muhammad Ramzan, Abida Rafiq, Jae Dong Chung, Seifedine Kadry, Yu-Ming Chu

**Affiliations:** 1 Department of Computer Science, Bahria University, Islamabad Campus, Islamabad, 44000 Pakistan; 2 Department of Mechanical Engineering, Sejong University, Seoul, 143-747 Korea; 3 Department of Mathematics and Computer Science, Faculty of Science, Beirut Arab University, Beirut, 115020 Lebanon; 4 Department of Mathematics, Huzhou University, Huzhou, 313000 People’s Republic of China; 5 Hunan Provincial Key Laboratory of Mathematical Modeling and Analysis in Engineering, Changsha University of Science and Technology, Changsha, 410114 People’s Republic of China

**Keywords:** Mathematics and computing, Mechanical engineering

## Abstract

The study of nanofluids is the most debated subject for the last two decades. Researchers have shown great interest owing to the amazing features of nanofluids including heat transfer and thermal conductivity enhancement capabilities. Having such remarkable features of nanofluids in mind we have envisioned a mathematical model that discusses the flow of nanofluid comprising Nickel-Zinc Ferrite-Ethylene glycol (Ni-ZnFe_2_O_4_–C_2_H_6_O_2_) amalgamation past an elongated curved surface with autocatalytic chemical reaction. The additional impacts added to the flow model are the heat generation/absorption with nonlinear thermal radiation. At the boundary, the slip and the convective conditions are added. Pertinent transformations are affianced to get the system of ordinary differential equations from the governing system in curvilinear coordinates. A numerical solution is found by applying MATLAB build-in function bvp4c. Graphical illustrations and the numerically computed estimates are discussed and analyzed properly. It is comprehended that velocity and temperature distributions have varied trends near and away from the curve when the curvature parameter is enhanced. Further, it is comprehended that the concentration field declines for both homogeneous and heterogeneous reaction parameters.

## Introduction

Nanofluids are characterized as a new type of nanotechnology centered fluids with enhanced thermal features, especially with regard to heat transfer. Nanofluids are the amalgamation of the metallic nanoparticles and some customary fluid like water or ethylene glycol. Choi and Eastman^1^ were the pioneer who floated the idea of nanofluids by inserting nanosized (< 100 nm) metallic particles into the usual fluid. Nanofluids are used to improve the thermal performance of microelectronics, microchips in computers, and fuel cells. Nanofluids are also handy in numerous engineering applications including heat exchanger, electronic devices’ cooling, reactors, biomedicine, transportation, and transformers’ cooling, etc.^2^. Maxwell^3^ proposed a model to examine the thermal conductivity effectiveness of nanofluids. This model includes the addition of spherical nanoparticles in the base fluid. Hamilton and Crosser^4^ suggested a model which is suitable for nanoparticles with non-spherical shape. Lately, Nadeem et al.^5^ numerically studied the hybrid nanofluid flow comprising two types of nanomaterials Copper and Aluminum Oxide immersed into water over a spongy exponentially stretched curved surface. It is determined in this study that nanofluids possess a higher heat transfer rate when compared with the ordinary fluid. The unsteady flow of the Sisko nanofluid with heat generation and thermal radiation past a curved surface is examined by Ali et al.^6^. It is comprehended here that the fluid velocity is enhanced for large curvature parameter. Khan et al.^7^ discussed numerically the dual solutions of a hybrid nanofluid flow containing silicon dioxide and Aluminum oxide as nanoparticles submerged into water over a curved surface near a stagnation point. It is witnessed here that thermal and velocity boundary layers are enriched when subject nanoparticles are inserted into the base fluid. The unsteady Casson Micropolar nanofluid flow in the region of a stagnation point over a curved surface is deliberated numerically by Amjad et al.^8^. The salient outcome of this study shows that higher estimates of the curvature parameter boost the fluid velocity and an opposite trend is noticed for the microrotation parameter. Some more recent studies featuring nanofluids may be found in^9–11^.

Over the last two decades, investigations emphasizing the boundary layer flow over the stretched surfaces has gained remarkable attention by the scientists and the engineers owing to its numerous uses in varied manufacturing and industrial processes. These procedures include liquid films in concentration processes, production of paper, designs of plastic wires and films, crystal glowing, glass blowing, food industries, coatings, drug delivery systems, paints, ceramics, and manufacturing of rubber sheets, etc. The pioneering work studying the fluid flow over a stretched surface was done by Crane^12^. Later, many researchers examined flows over stretching surfaces under different configurations. Gutpa and Gupta^13^ examined the fluid flow over the spongy surface. Hydromagnetic flow past a stretching surface is assumed by Charabakti and Gupta^14^. Anderson et al.^15^ examined Power-law fluid flow under the impact of magnetic forces past a linearly stretching sheet. An Oldroyd-B fluid flow under the influence of generation or absorption of heat is depicted by Hayat et al.^16^. Sajid et al.^17^ investigated the first time the fluid flow through a straining/stretching surface with a curved shape applying curvilinear coordinates. The impact of the thermal stratification phenomenon in the ferromagnetic fluid over a stretched sheet is scrutinized by Muhammad et al*.*^18^. Ramzan and Yousaf^19^ determined the elastic viscid nanofluid flow in view of Newtonian heating past a two-dimensional straining sheet. Sanni et al.^20^ proposed a computational analysis on the viscous fluid flow past a stretchable curved surface. Hussain et al.^21^ analyzed the flow of Jeffery nano-fluid along with radiation effects past an exponentially stretching sheet. Some recent research work on different types of fluid flows over a stretching surface is given at^22–26^.

Homogeneous-heterogeneous reactions are entangled in the various bio-chemical reacting systems e.g., biochemical systems and hydrolysis. The association between homogenous and heterogeneous reactions is quite complicated. Some reactions can continue steadily without a catalyst. Homogenous and heterogeneous reactions are unpredictable in the case of reactant species creation and are consumed on the surface of the catalyst. These types of reactions are witnessed in fog formation, processing and diffusion of food, polymer production, hydro-metallurgical industry, and ceramics. Homogenous and heterogeneous reactions impact in a viscous fluid flow past an extended surface is investigated by Merkin^27^. It is observed here that heterogeneous reaction appears on the surface of the catalyst and homogenous reaction for cubic autocatalysis. Chaudhary and Markin^28^ reported the same diffusivities of homogenous and heterogeneous reactions. Bachok et al.^29^ determined the homogenous and heterogeneous reactions in stretching fluid flow. Stretching viscous fluid flow with homogenous-heterogeneous reactions is assumed by Khan and Pop^30^. Recently, Saif et al.^31^ analyzed analytically the boundary layer flow over a nonlinear curved stretched surface in the presence of autocatalytic reactions and the convective boundary condition at the surface using the Homotopy Analysis Method. The outcome of the analysis revealed that the fluid temperature is enhanced for large estimates of Biot number, nevertheless, an opposing behavior is noted for autocatalytic chemical reactions. The flow of the nanofluid comprising nanomaterials with autocatalytic reactions and second-order boundary condition accompanied by entropy minimization analysis is numerically explored by Muhammad et al.^32^. It is disclosed in this study that the fluid velocity is diminished for decreasing value of the slip condition. The Peristaltic MHD hybrid Carreau nanofluid flow comprising copper and silver nanoparticles with autocatalytic reactions is studied by Bibi and Xu^33^. The salient outcome of this model is that the increasing values of the Weissenberg and the Hartmann numbers affect the fluid velocity. Homogenous and heterogeneous reactions are also conferred through the several studies given at^34–41^.

From the above literature review, it is noted that very little work is available on the fluid flow over curved surfaces as compared to linear/non-linear/exponential stretching surfaces. Further, this difference gets narrower if we speak about the study of nanofluid with homogenous and heterogeneous reactions past a curved surface. Our aim in this exploration is to numerically analyze the flow of nanofluid containing ferromagnetic nanoparticles i.e., Nickel zinc ferrite (${\text{NiZnFe}}_{2} {\text{O}}_{4}$) and the base fluid, ethylene glycol (${\text{C}}_{2} {\text{H}}_{6} {\text{O}}_{2}$) with impacts of autocatalytic reactions, heat generation/absorption, and nonlinear thermal radiation. The analysis is supported by the slip and convective conditions at the boundary of the curved surface that also boosts the novelty of the envisioned mathematical model. The numerical solution of the proposed problem is attained by invoking $bvp4c$ from MATLAB, and characteristics of all arising parameters are discussed thoroughly by keeping their physical justification in mind.

## Mathematical modeling

**Figure 1:**
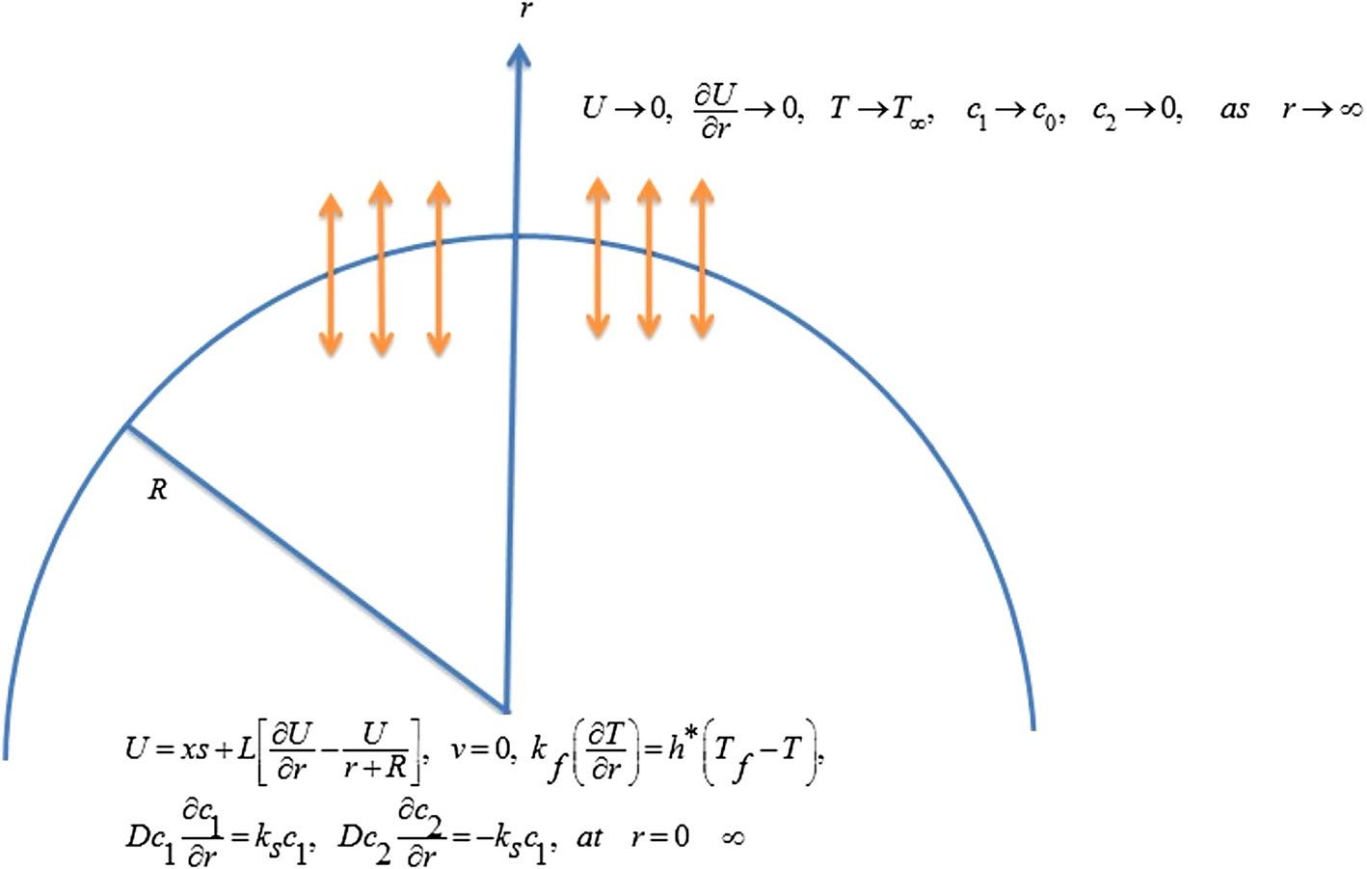
Geometry of the envisioned nanofluid flow.

Consider an incompressible 2D nanofluid flow over a spiral steady stretched sheet coiled in a semi-circle of the radius $R^{*}$. The sheet is stretched with the velocity $U = u_{w}$ in the *x-*direction. The induced magnetic field $B_{0}$ is enforced in *r-*direction *i.e.,* perpendicular to the fluid flow. The temperature *T* of the sheet is kept invariable while $T_{\infty }$ is the ambient temperature of the fluid. The heat transfer process is examined through heat absorption/generation influences (Fig. 1).

Homogenous and heterogeneous reactions with two synthesized species $S_{1}$ and $S_{2}$ are considered. For cuboid autocatalysis, the homogenous reaction can be defined as^28^:

1 $$S_{1} + 2S_{2} \to 3S_{2} ,\quad rate = k_{c} c_{1} c_{2}^{2} ,$$

whereas the heterogeneous reaction on the reactant surface is depicted as^28^:

2 $$S_{1} \to S_{2} ,\quad rate = k_{s} c_{1} .$$

Here, $c_{1} ,c_{2}$ are the concentrations for the chemical species $S_{1}$ and $S_{2}$ whereas rate constants are $k_{c}$ and $k_{s}$. The assumed system of equations comprising continuity, momentum, energy, and homogeneous–heterogeneous reactions is governed by the subsequent boundary layer equations^9^.

3 $$R^{*} \left( {\frac{\partial U}{{\partial x}}} \right) + \frac{\partial }{\partial r}\left\{ {\left( {r + R^{*} } \right)v} \right\} = 0,$$

4 $$\frac{{U^{2} }}{{r + R^{*} }} = \frac{1}{{\rho_{nf} }}\left( {\frac{\partial p}{{\partial r}}} \right),$$

5 $$\begin{aligned} & v \left( {\frac{\partial U}{{\partial r}}} \right) + \frac{{UR^{*} }}{{r + R^{*} }}\left( {\frac{\partial U}{{\partial x}}} \right) + \frac{Uv}{{r + R^{*} }} = - \frac{1}{{\rho_{nf} }}\left( {\frac{{R^{*} }}{{R^{*} + r}}} \right)\left( {\frac{\partial p}{{\partial x}}} \right) \\ & \quad + \frac{{\mu_{nf} }}{{\rho_{nf} }}\left\{ {\frac{{\partial^{2} U}}{{\partial r^{2} }} + \frac{1}{{R^{*} + r}}\left( {\frac{\partial U}{{\partial r}}} \right) - \frac{U}{{\left( {R^{*} + r} \right)^{2} }}} \right\} - \frac{\sigma }{{\rho_{nf} }}B_{0}^{2} U, \\ \end{aligned}$$

6 $$\begin{aligned} & v\left( {\frac{\partial T}{{\partial r}}} \right) + \left( {\frac{{UR^{*} }}{{R^{*} + r}}} \right)\left( {\frac{\partial T}{{\partial x}}} \right) = \alpha_{nf} \left\{ {\frac{{\partial^{2} T}}{{\partial r^{2} }} + \frac{1}{{R^{*} + r}}\left( {\frac{\partial T}{{\partial r}}} \right)} \right\} \\ & \quad \frac{{Q_{0} }}{{(\rho C{}_{p})_{nf} }}\left( {T_{\infty } - T} \right) + \frac{1}{{(\rho C_{p} )_{nf} }}\left( {\frac{1}{{r + R^{*} }}} \right)\frac{\partial }{\partial r}\left( {r + R^{*} } \right)q_{r} , \\ \end{aligned}$$

7 $$v\left( {\frac{{\partial c_{1} }}{\partial r}} \right) + \frac{{R^{*} U}}{{R^{*} + r}}\left( {\frac{{\partial c_{1} }}{\partial x}} \right) = Dc_{1} \left\{ {\frac{{\partial^{2} c_{1} }}{{\partial r^{2} }} + \frac{1}{{R^{*} + r}}\left( {\frac{{\partial c_{1} }}{\partial r}} \right)} \right\} - k_{c} c_{1} c_{2}^{2} ,$$

8 $$v\left( {\frac{{\partial c_{2} }}{\partial r}} \right) + \frac{{R^{*} U}}{{R^{*} + r}}\left( {\frac{{\partial c_{2} }}{\partial x}} \right) = Dc_{2} \left\{ {\frac{{\partial^{2} c_{2} }}{{\partial r^{2} }} + \frac{1}{{R^{*} + r}}\left( {\frac{{\partial c_{2} }}{\partial r}} \right)} \right\} + k_{c} c_{1} c_{2}^{2} .$$

With the subject conditions:

9 $$\begin{aligned} & U = xs + L\left[ {\frac{\partial U}{{\partial r}} - \frac{U}{{r + R^{*} }}} \right],\;v = 0,\;k_{f} \left( {\frac{\partial T}{{\partial r}}} \right) = h^{*} \left( {T_{f} - T} \right), \\ & Dc_{1} \frac{{\partial c_{1} }}{\partial r} = k_{s} c_{1} ,\;Dc_{2} \frac{{\partial c_{2} }}{\partial r} = - k_{s} c_{1} ,\;{\text{at}}\;r = 0, \\ & U \to 0,\;\frac{\partial U}{{\partial r}} \to 0,\;T \to T_{\infty } ,\;c_{1} \to c_{0} ,\;c_{2} \to 0,\;{\text{as}}\;r \to \infty . \\ \end{aligned}$$

Invoking the following transformations:

10 $$\begin{aligned} \xi = & \sqrt {\frac{s}{{v_{f} }}} r,\;p = \rho_{f} s^{2} x^{2} P(\xi ),\;T = T_{\infty } \left( {1 + \left( {\theta_{w} - 1} \right)\theta } \right),\;U = sxf^{{\prime}} \left( \xi \right), \\ c_{1} = & c_{0} \varphi (\xi ),\;c_{2} = c_{0} g(\xi ),\;v = - \frac{{R^{*} }}{{r + R^{*} }}\sqrt {sv_{f} } f\left( \xi \right). \\ \end{aligned}$$

After using boundary layer approximation and invoking here, Eq. (3) is automatically equated and Eqs. (4)–(10) take the form:

11 $$P^{\prime} = \left( {1 - \Phi + \Phi \frac{{\rho_{s} }}{{\rho_{f} }}} \right)\frac{{f^{{\prime}{2}} }}{{\xi + K_{1} }}.$$

12 $$\begin{aligned} & \frac{1}{{\left( {1 - \Phi + \Phi \frac{{\rho_{s} }}{{\rho_{f} }}} \right)}}\frac{{2K_{1} }}{{\xi + K_{1} }}P = \frac{1}{{(1 - \Phi )^{{\frac{25}{{10}}}} \left( {1 - \Phi + \Phi \frac{{\rho_{s} }}{{\rho_{f} }}} \right)}}\left( {f^{{{\prime \prime \prime }}} - \frac{{f^{{\prime}} }}{{(\xi + K_{1} )^{2} }} + \frac{{f^{{\prime \prime }} }}{{\xi + K_{1} }}} \right) \\ & \quad - \frac{{K_{1} }}{{K_{1} + \xi }}ff^{{\prime \prime }} + \frac{{K_{1} }}{{(K_{1} + \xi )^{2} }}ff^{{\prime}} - \frac{Ha}{{\left( {1 - \Phi + \Phi \frac{{\rho_{s} }}{{\rho_{f} }}} \right)}}f^{{\prime}} . \\ \end{aligned}$$

13 $$\begin{aligned} & \frac{1}{\Pr }\left\{ {\frac{{k_{nf} }}{{k_{f} }} + R_{d} \left( {1 + \theta (\theta_{w} - 1)} \right)^{3} } \right\}\left( {\theta^{{\prime \prime }} + \frac{1}{{K_{1} + \xi }}\theta^{{\prime}} } \right) + \left( {1 - \Phi + \Phi \frac{{(\rho C_{p} )_{s} }}{{(\rho C_{p} )_{f} }}} \right)\left( {\frac{{K_{1} }}{{\xi + K_{1} }}f\theta^{{\prime}} } \right) \\ & \quad - \lambda_{1} \theta + \frac{3}{\Pr }R_{d} \left( {1 + (\theta_{w} - 1)\theta } \right)^{2} \theta^{{{\prime}2}} \left( {\theta_{w} - 1} \right) = 0. \\ \end{aligned}$$

14 $$\frac{1}{{S_{c} }}\left( {\psi^{\prime\prime} + \frac{1}{{\xi + K_{1} }}\psi^{\prime}} \right) + \frac{{K_{1} }}{{\xi + K_{1} }}f\psi^{\prime} - k_{1} \psi g^{2} = 0.$$

15 $$\frac{\delta }{{S_{c} }}\left( {g^{\prime\prime} + \frac{1}{{\xi + K_{1} }}g^{\prime}} \right) + \frac{{K_{1} }}{{\xi + K_{1} }}fg^{\prime} + k_{1} \psi g^{2} = 0.$$

16 $$\begin{array}{*{20}l} {f(\xi ) = 0,f^{{\prime}} (\xi ) = 0,f^{{\prime}} (\xi ) = 1 + K^{*} \left[ {f^{{\prime\prime }} (\xi ) - f^{{\prime}} (\xi )} \right],} \hfill \\ {\theta^{{\prime}} (\xi ) = Bi(1 - \theta (\xi )),\psi^{{\prime}} (\xi ) = k_{2} \psi (\xi ),\delta g^{{\prime}} (\xi ) = - k_{2} \psi (\xi ),} \hfill \\ \end{array} \quad {\text{at}}\;\xi = 0,$$

17 $$f^{\prime}(\infty ) \to 0,f^{\prime\prime}(\infty ) \to 0,\theta (\infty ) \to 0,\quad {\text{as}}\;\xi \to \infty .$$

The arising parameters are mathematically defined as:

18 $$\begin{aligned} K_{1} = & R^{*} \sqrt {\frac{s}{{v_{f} }}} ,\;Bi = h^{*} \frac{{\sqrt {\frac{{v_{f} }}{s}} }}{{k_{f} }},\;S_{c} = \frac{{v_{f} }}{{Dc_{1} }},\;R_{d} = \frac{{16\sigma^{*} T_{\infty }^{3} }}{{3k^{*} k}},\;\Pr = \frac{{v_{f} }}{\alpha },\;K^{*} = L\sqrt {\frac{s}{{v_{f} }}} . \\ \lambda_{1} = & \frac{{Q_{0}^{*} }}{{s(\rho C_{p} )_{f} }},\;Ha = \sigma \frac{{B_{0}^{2} }}{{s\rho_{f} }},\;k_{1} = c_{0}^{2} \frac{{k_{c} }}{s},\;k_{2} = \frac{{k_{s} \sqrt {\frac{{v_{f} }}{s}} }}{{Dc_{1} }},\;\delta = \frac{{Dc_{2} }}{{Dc_{1} }}. \\ \end{aligned}$$

Eliminating pressure from Eqs. (11)–(12), we get

19 $$\begin{aligned} & f^{iv} + \frac{{2f^{{{\prime\prime \prime }}} }}{{K_{1} + \xi }} - \frac{{f^{{\prime\prime }} }}{{(K_{1} + \xi )^{2} }} + \frac{{f^{{\prime}} }}{{(K_{1} + \xi )^{3} }} + \left( {1 - \Phi } \right)^{2.5} \left( {1 - \Phi + \Phi \frac{{\rho_{s} }}{{\rho_{f} }}} \right) \\ & \quad \left[ \begin{gathered} \frac{{K_{1} }}{{(\xi + K_{1} )^{2} }}\left( {f^{{{\prime}2}} - ff^{{\prime\prime }} } \right) \hfill \\ - \frac{{K_{1} }}{{\xi + K_{1} }}\left( {f^{{\prime\prime }} f^{{\prime}} - ff^{{{\prime\prime \prime }}} } \right) - \frac{{K_{1} }}{{(\xi + K_{1} )^{3} }}ff^{{\prime}} \hfill \\ \end{gathered} \right] - (1 - \Phi )^{2.5} Ha\left( {\frac{{f^{{\prime}} }}{{\xi + K_{1} }} + f^{{\prime\prime }} } \right) = 0 \\ \end{aligned}$$

Table 1 characterizes the physical characteristics of Ethylene glycol- Nickel-zinc ferrite **(**Ni-ZnFe_2_O_4_–C_2_H_6_O_2_).

**Table 1:** Mathematical values of physical properties $C_{p}$, $\rho$, and *k* for Ethylene glycol and Nickel-zinc ferrite (Ni-ZnFe_2_O_4_–C_2_H_6_O_2_)^9^.

Material	C_*p*_ (J kg^−1^ k^−1^)	*ρ* (kg m^−3^)	*k* (W m^−1^ K^−1^)
Ethylene–glycol C_2_H_6_O_2_	2382.0	1116.6	0.2490
Nickel-zinc ferrite (NiZnFe_2_O_4_)	710.0	4800.0	6.3

Thermophysical properties in the mathematical form are given as^42^:

20 $$\mu_{nf} = \frac{{\mu_{f} }}{{(1 - \Phi )^{{\frac{25}{{10}}}} }},$$

21 $$\alpha_{nf} = \frac{{k_{nf} }}{{(\rho C_{p} )_{nf} }},$$

22 $$\rho_{nf} = (1 - \Phi )\rho_{f} + \Phi \rho_{s} .$$

23 $$(\rho C_{p} )_{nf} = \Phi (\rho C_{p} )_{s} + (1 - \Phi )(\rho C_{p} )_{f} .$$

24 $$\frac{{k_{nf} }}{{k_{f} }} = \frac{{k_{s} + 2k_{f} + 2\Phi (k_{s} - k_{f} )}}{{k_{s} + 2k_{f} - 2\Phi (k_{s} - k_{f} )}}.$$

When the diffusion coefficients $Dc_{1} = Dc_{2} ,$ then $\delta = 1,$ we have

25 $$\psi (\xi ) + g(\xi ) = 1.$$

Then Eqs. (14) and (15) become

26 $$\frac{1}{{S_{c} }}\left( {\psi^{\prime\prime} + \frac{1}{{\xi + K_{1} }}\varphi^{\prime}} \right) + \frac{{K_{1} }}{{\xi + K_{1} }}f\psi^{\prime} - k_{1} \psi \left( {1 - \psi } \right)^{2} = 0,$$

with boundary conditions

27 $$\psi^{{\prime}} (0) = k_{2} \psi (0),\quad \psi (0) \to 1.$$

Along *x*-direction, Nusselt number $(Nu_{x} ),$ surface drag force coefficient $(C_{f} )$ is:

28 $$Nu_{x} = \frac{{xq_{r} }}{{k_{f} \left( {T_{f} - T_{\infty } } \right)}},\quad C_{f} = \frac{{2\tau_{rx} }}{{u_{w}^{2} \rho }},$$

29 $$(q_{r} )_{w} = q_{w} + \left( {\frac{\partial T}{{\partial r}}} \right),\quad \tau_{rx} = \mu_{nf} \left( {\frac{\partial U}{{\partial r}} - \frac{U}{{r + R^{*} }}} \right),\;{\text{at}}\;r = 0$$

Nusselt number and surface drag force in the dimensionless form are appended as follows:

30 $$\begin{aligned} Nu_{x} \left( {{\text{Re}}_{x} } \right)^{{ - \frac{1}{2}}} = & - \left\{ {\frac{{k_{nf} }}{{k_{f} }} + R_{d} \left[ {1 + \left( {\theta_{w} - 1} \right)\theta (0)} \right]^{3} } \right\}\theta (0), \\ \frac{1}{2}C_{f} \left( {{\text{Re}}_{x} } \right)^{\frac{1}{2}} = & f^{\prime\prime } (0) - \frac{{f^{\prime } (0)}}{{K_{1} }}. \\ \end{aligned}$$

The local Reynolds number is expressed as ${\text{Re}}_{x} = \frac{{sx^{2} }}{{v_{f} }}.$

## Results and discussion

**Figure 2:**
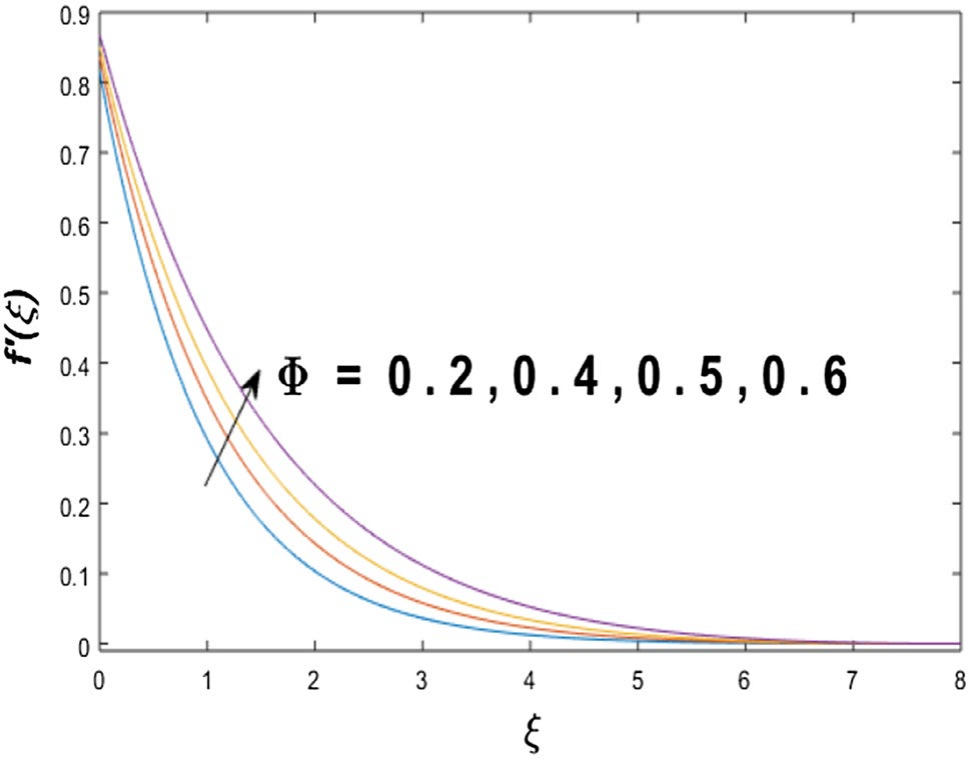
Variations of $\Phi$ to $f^{\prime}(\xi )$.

**Figure 3:**
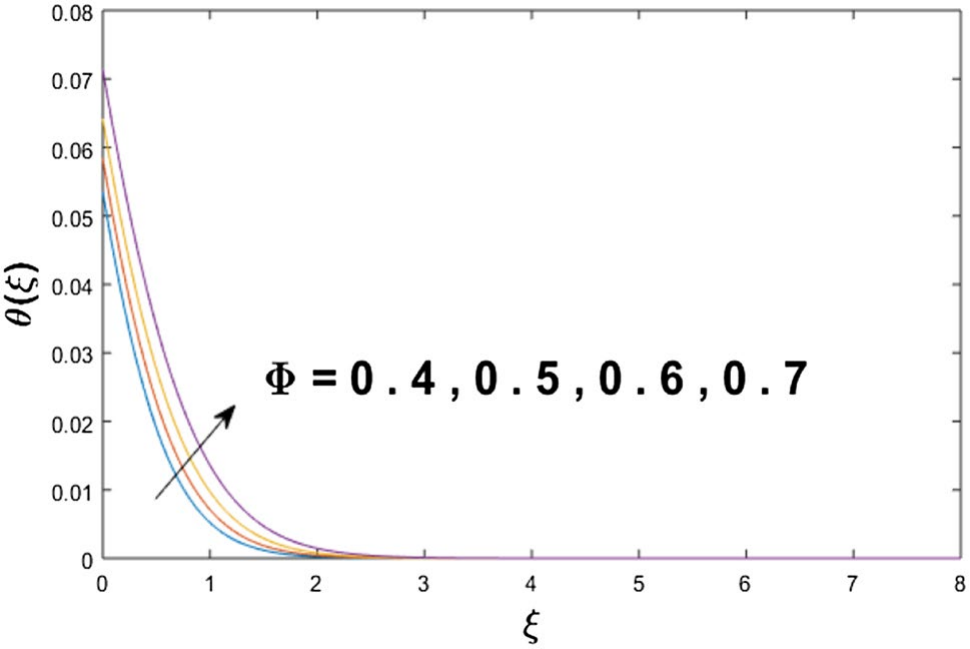
Variations of $\Phi$ to $\theta (\xi )$.

**Figure 4:**
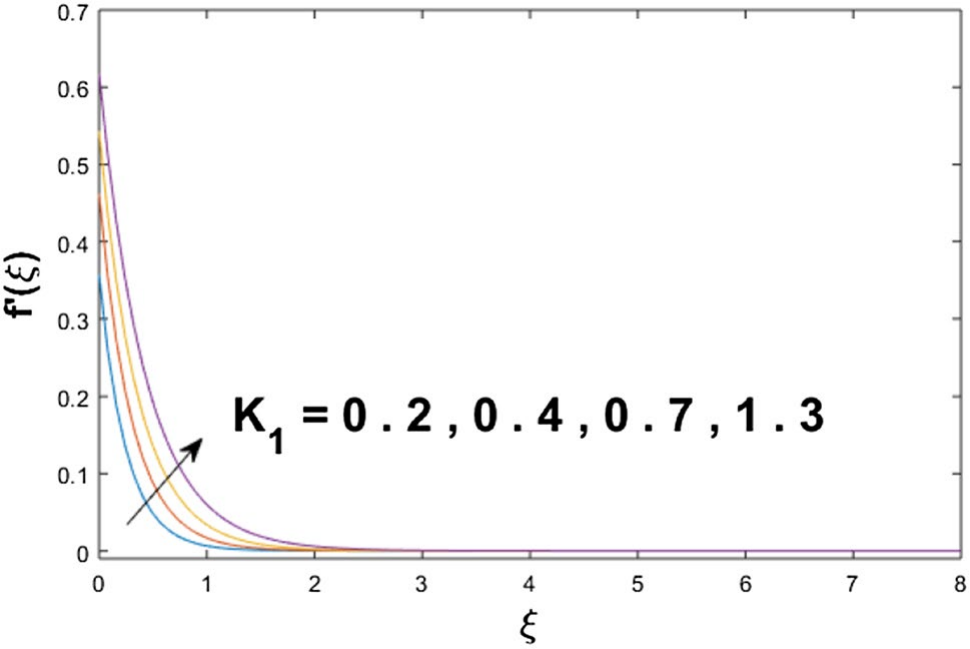
Variations of $K_{1}$ to $f^{\prime}(\xi )$.

**Figure 5:**
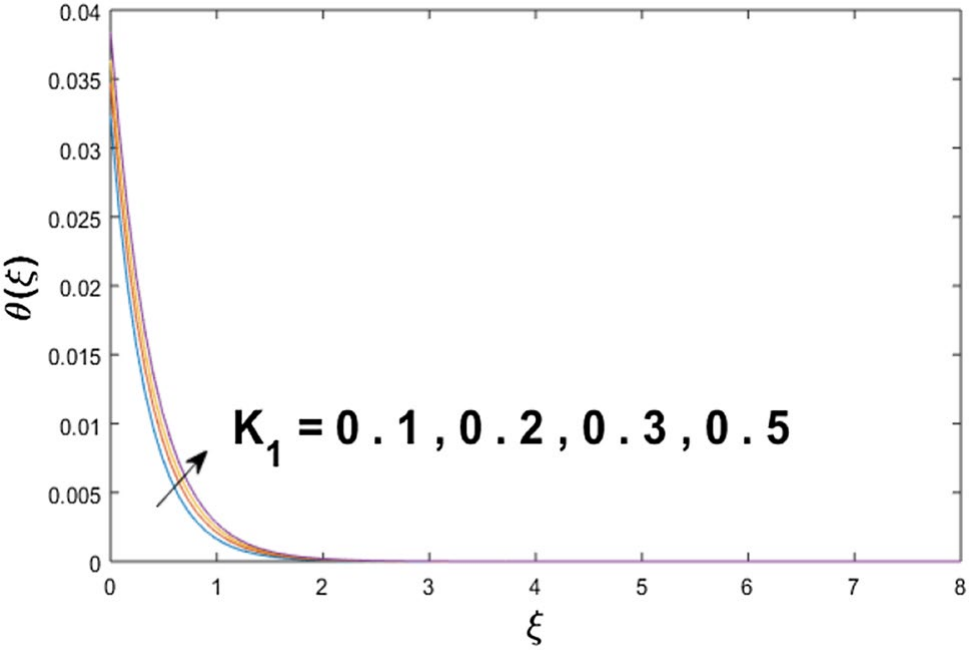
Variations of $K_{1}$ to $\theta (\xi )$.

**Figure 6:**
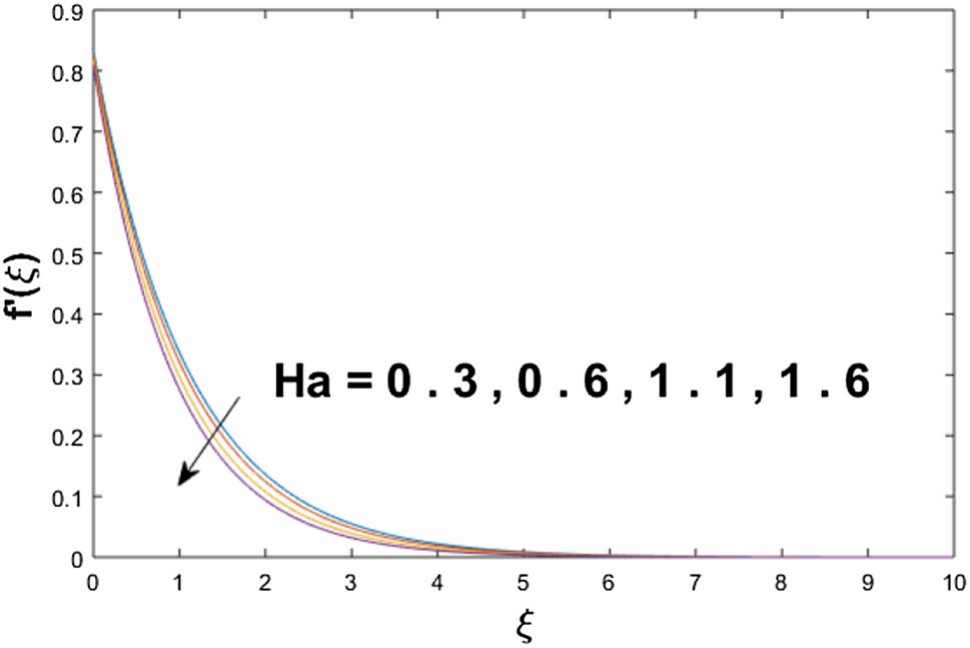
Variations of *Ha* to $f^{\prime}(\xi )$.

**Figure 7:**
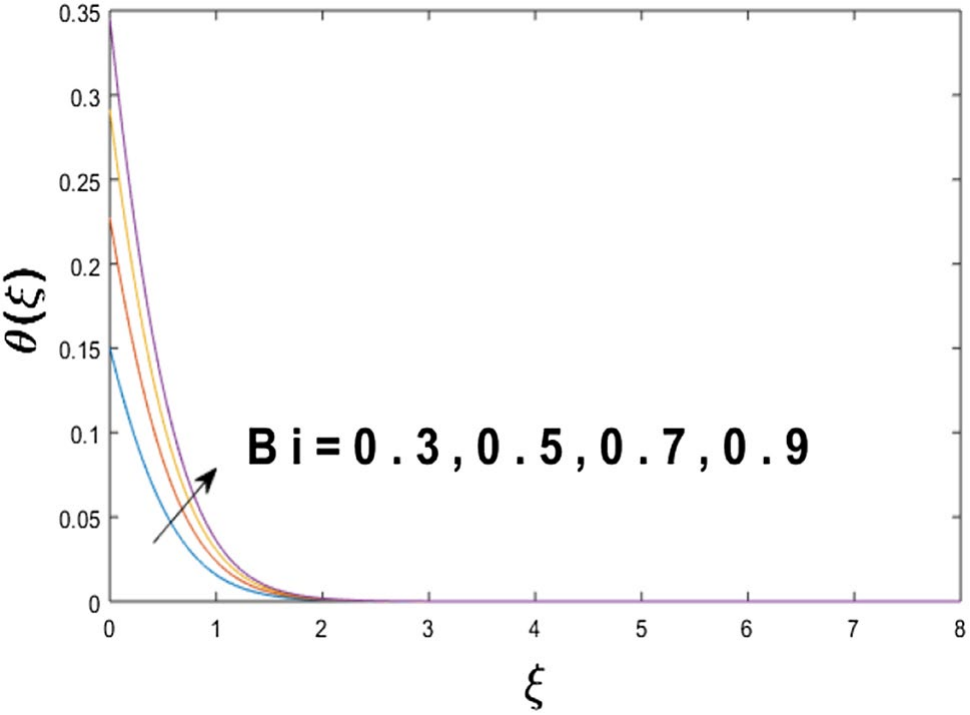
Variations of *Bi* to $\theta (\xi )$.

**Figure 8:**
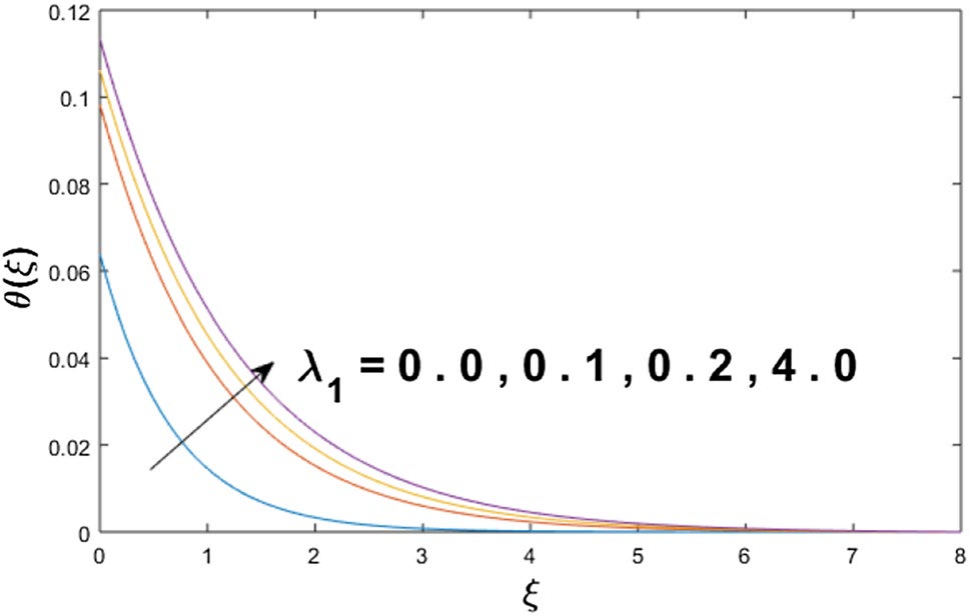
Variations of $\lambda_{1}$ to $\theta (\xi )$.

**Figure 9:**
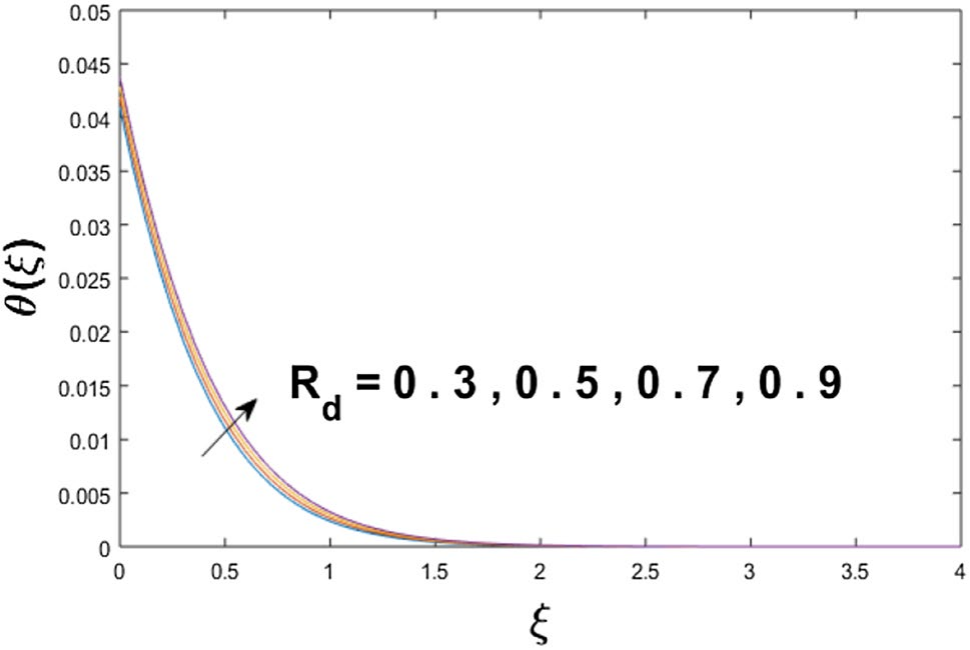
Variations of $R_{d}$ to $\theta (\xi )$.

**Figure 10:**
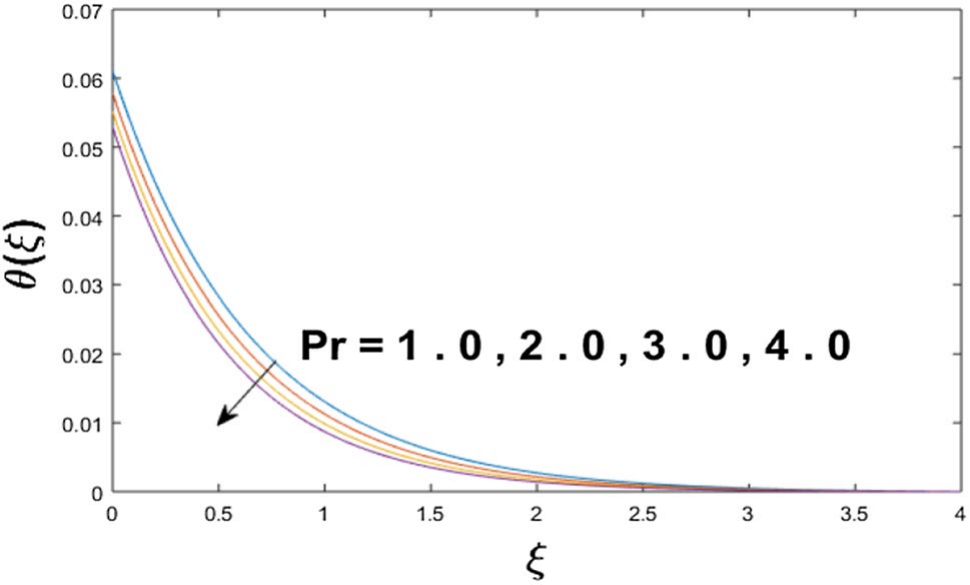
Variations of Pr to $\theta (\xi )$.

**Figure 11:**
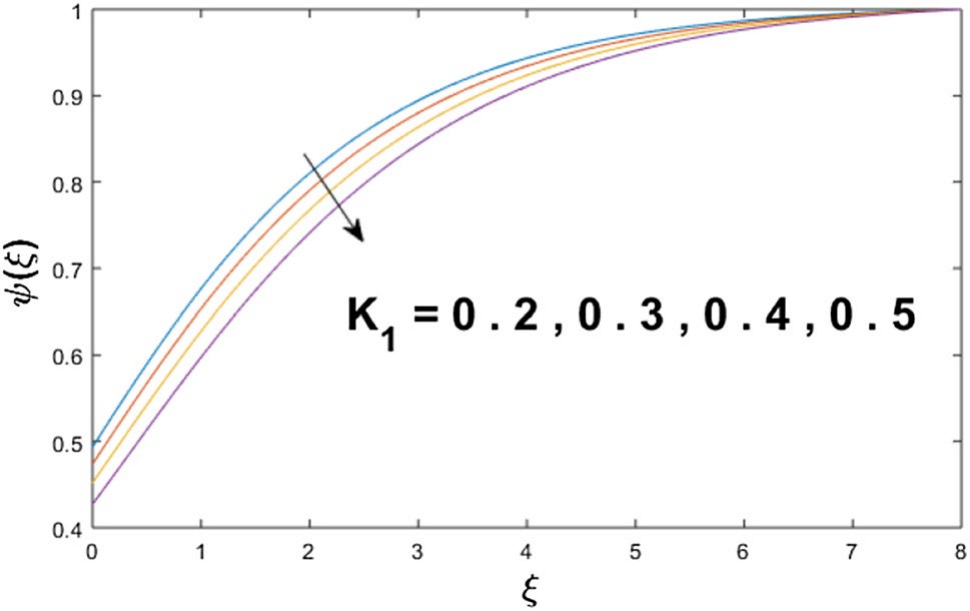
Variations of $K_{1}$ to $\psi (\xi )$.

**Figure 12:**
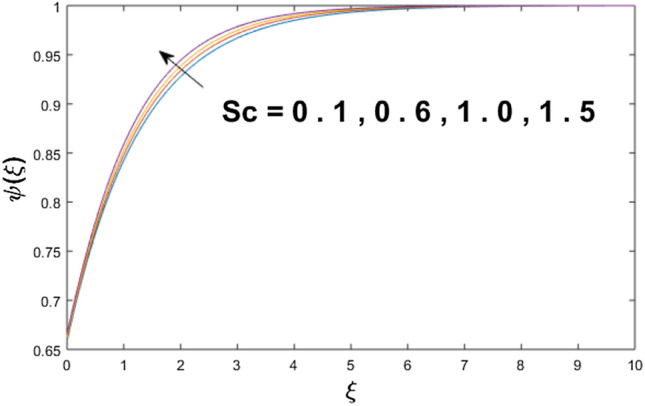
Variations of $S_{c}$ to $\psi (\xi )$.

**Figure 13:**
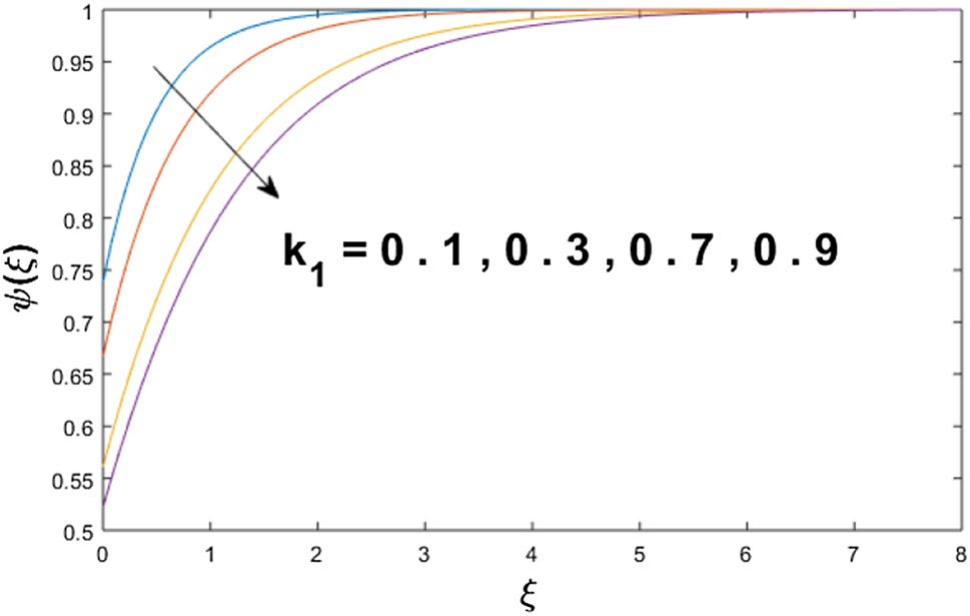
Variations of $k_{1}$ to $\psi (\xi )$.

**Figure 14:**
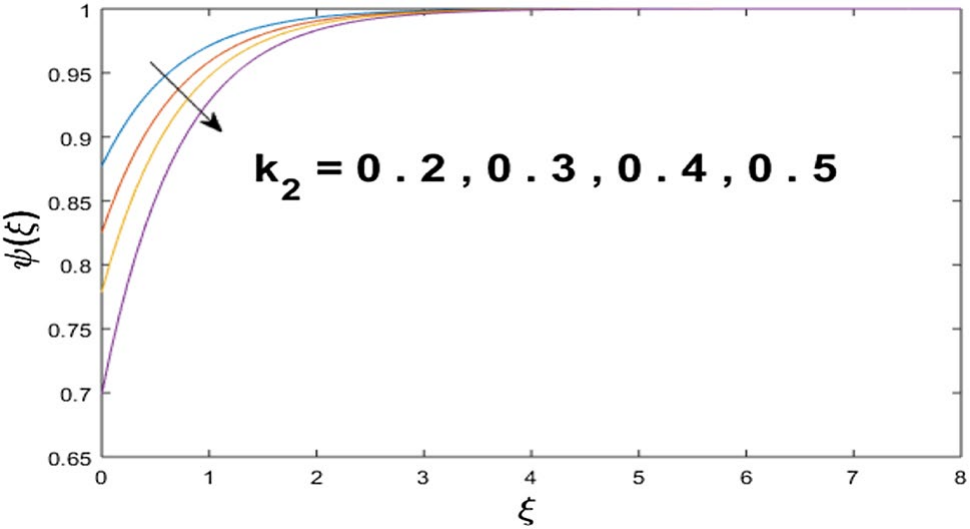
Variations of $k_{2}$ to $\psi (\xi )$.

The velocity profile, temperature distribution, and rate of heat transfer are characterized by solving Eqs. (12), (13), (19), (26) subject to boundary conditions (16), (17), and (27), and are solved by using MATLAB built-in function bvp4c. This particular section is dedicated to envisioning the impact of distinct parameters, on the velocity $$f^{\prime}(\xi )$$, temperature $$\theta (\xi )$$, and concentration $$\psi (\xi )$$ profiles. The parameters with their fixed values are defined as $$\Pr = K_{1} = 10,S_{c} = 0.5,R_{d} = 1.5,\lambda_{1} = 10.5,k_{1} = Bi = k_{2} = 0.1$$, $$\Phi = Ha = 0.1$$. Figures 2 and 3 are illustrated to see the effects of volume fraction $$\Phi$$ on the velocity and temperature fields respectively. The magnitude of fluid velocity and temperature field upsurge with increasing values of $$\Phi$$. For large values of $$\Phi$$, the momentum, and the boundary layer thicknesses are getting thicker. The nanoparticles volume fraction and convective flow are in direct proportionate with each other. So, an increase in the volume fraction boosts the velocity of the fluid flow. In Fig. 3, the increase in the temperature is because of the thermal conductivity of nanoparticles that is enhanced with growing estimates of nanoparticles volume fraction. This why the high temperature of the fluid is witnessed for large estimates of nanoparticle volume fraction. Figure 4 is drawn to check the inspiration of the curvature parameter $$K_{1}$$ on the velocity profile $$f^{{\prime}} (\xi )$$. The velocity of the fluid raises with large values of $$K_{1}$$. The surface radius increases for higher estimates of curvature parameter $$K_{1}$$, which enhances the fluid velocity. Figure 5 demonstrates the behavior of the temperature field for distinct estimates of $$K_{1}$$. It is observed that the higher values of $$K_{1}$$ initiates an increase in temperature and the thermal boundary layer. An increase in heat transport is witnessed as values of the curvature parameter is improved. Figure 6 elucidates the influence of the magnetic parameter *M* on velocity distribution $$f^{^{\prime}} (\xi )$$. A decline in the magnitude of velocity is dependent on the enhancement of Hartman's number *Ha*. This is because the external magnetic field acts as a resistive force (Lorentz force) to the fluid velocity. Figures 7 and 8 illustrate the characteristic of *Bi* (Biot number) and heat source (generation of heat or absorption) parameter $$\lambda_{1}$$ on temperature distribution $$\theta (\xi )$$, respectively. Figure 7 depicts that the convective heat transfer coefficient augments for higher values of *Bi* and the temperature eventually boosted. Figure 8 demonstrates the temperature field affected by $$\lambda_{1}$$. So, augmentation in $$\lambda_{1}$$ leads to an increment in the temperature and thickness of the thermal boundary layer. Figure 9 depicts the change of radiation parameter $$R_{d}$$ on the dimensionless temperature profile $$\theta (\xi )$$. Augmentation in temperature profile is noted for increasing values of $$R_{d}$$. Substantially, the conduction effects are enhanced for cumulative values of $$R_{d}$$, hence; increase in temperature of the fluid can be noticed. Figure 10 displays the effect of the Prandtl number Pr on temperature distribution. It is obvious from this figure that the temperature profile declines with the increasing values of Pr, as the thermal diffusivity and Prandtl number are associated reciprocally. As the increase in Pr causes a reduction in temperature as well as in boundary layer thickness. The effect of the curvature parameter $$K_{1}$$ on concentration distribution is depicted in Fig. 11. It shows that the concentration field reduces for higher values of the curvature parameter $$K_{1}$$$$.$$ Large estimates of the curvature parameter increase the space for the collision of the molecules. Thus, decreasing the concentration of the fluid. Figure 12 portrays a decline in concentration $$\psi (\xi )$$ with increasing estimates of the Schmidt number. The relation between mass diffusivity and viscosity is called the Schmidt number $$S_{c}$$$$.$$ Then mass diffusivity declines with increasing Schmidt number. Consequently, a reduction in the concentration of fluid is seen. Figure 13 specifies that the concentration field $$\psi (\xi )$$ decreases with the high strength of the homogenous reaction parameter $$k_{1}$$. Actually, the reactants are consumed during the homogenous reaction. Thus, a decline in concentration is observed. The concentration profile for a larger strength of heterogeneous reaction parameter $$k_{2}$$ is explained in Fig. 14. When $$k_{2}$$ increases, the concentration profile decreases because of a reduction in diffusion.

Table 2 shows the impact of prominent parameters on the Skin drag coefficient and Nusselt number. The drag strength coefficient rises for large values of *Ha* (Hartman number), $\Phi$ (solid volume fraction of nanofluid) and $K_{1}$ (curvature parameter). A gradual reduction in Nusselt number is noted for higher values of $K_{1}$. Both the Nusselt number and Skin friction coefficient raises for larger values of solid volume fraction $\Phi$ of nanofluid. For high radiation parameter $R_{d}$, Biot number $Bi,\lambda_{1}$ and $\theta_{w}$ heat transmission rate enhance and no influence on the Skin drag coefficient.

**Table 2:** Values of Nusselt number $Nu(Re_{x} )^{ - 1/2}$ and Skin drag coefficient $- \frac{1}{2}C_{f} (Re_{x} )^{\frac{1}{2}}$.

$K_{1}$	$Ha$	$\Phi$	$Bi$	$R_{d}$	$\lambda_{1}$	$\theta_{w}$	$- \frac{1}{2}C_{f} (Re_{x} )^{\frac{1}{2}}$	$Nu(Re_{x} )^{ - 1/2}$
**0.8**	0.4	0.5	0.7	0.5	0.5	0.5	1.9567	0.3221
**1.0**							2.0963	0.3215
**1.3**							2.1979	0.3210
**1.5**							2.2344	0.3208
**0.8**	0.1						1.9369	0.3221
	0.3						1.9501	0.3221
	0.5						1.9633	0.3221
	1.0						1.9960	0.3221
	0.1	0.02					0.3837	0.1013
		0.2					0.6297	0.1577
		0.5					1.9369	0.3221
		0.5	0.7				–	1.7136
			1.0				–	2.1923
			1.5				–	2.8072
			2.0	0.1			–	3.2456
				0.3			–	3.2582
				0.5			–	3.2706
				0.7	0.7		–	3.4474
					0.9		–	3.5804
					1.0		–	3.6385
					0.7	0.5	–	3.4474
						0.7	–	3.4319
						0.9	–	3.4075

### Final remarks

In this study, we have studied the flow of the nanofluid comprising an amalgamation of the Nickel-Zinc ferrite and Ethylene glycol (Fe_2_O_4_–C_2_H_6_O_2_) past an elongated curved surface with the autocatalytic chemical reactions. The associated effects considered in the exploration are the heat generation/absorption and nonlinear thermal radiation with slip and convective conditions are taken at the boundary. The numerical solution of the envisaged problem is obtained via the bvp4c method, a MATLAB software tool. This fluid model is unique as it comprises the combined impacts of autocatalytic chemical reaction with heat generation/absorption and nonlinear thermal radiation with slip and convective conditions. No study so far has been carried out that discusses the combined impacts of the aforementioned impacts. The key observations of the presented model are:

- The autocatalyst chemical reactions affect the fluid concentration significantly.
- The fluid temperature is enhanced for growing estimates of Biot number, heat generation parameter, and solid volume fraction.
- The impacts of the solid volume fraction and the Hartmann number are opposite on the fluid velocity.
- The fluid concentration is augmented for large estimates of the radiation parameter.
- The Schmidt and the Prandtl numbers lower the fluid concentration.
- The impact of the Surface drag force is strengthed for growing values of the Hartmann and Biot numbers.
- The rate of heat transfer is augmented when numerical estimates of the radiation parameter and Biot number.

### Future possibilities

The subject manuscript may be extended to a hybrid nanofluid form with varied nanomaterials and fluids. The melting heat boundary conditions may also be adopted.

## List of symbols

$B_{0}$: Magnetic field strength
$$Bi$$: Biot number
$C_{f}$: Surface drag force
$c_{1} ,c_{2}$: Concentration of chemical species
$$C_{p}$$: Specific heat
$$Dc_{1} ,Dc_{2}$$: Diffusion coefficient
$f$: Dimensionless stream function
$$Ha$$: Magnetic parameter
$$k$$: Thermal conductance
$k_{f}$: Fluid thermal conductivity
$k_{nf}$: Nanofluid thermal conductivity
$K^{*}$: Dimensionless slip parameter
$k_{1}$: Strength of homogenous reaction
$$k_{c} ,k_{s}$$: Rate constants
$k_{2}$: Strength of heterogeneous reaction
$$K_{1}$$: Curvature parameter
$L$: Value of slip parameter
$$Nu_{x}$$: Nusselt number
$$P$$: Fluid pressure
$$\Pr$$: Prandtl number
$Q_{0}$: Heat generation/absorption volumetric rate
$q_{r}$: Nonlinear radiative heat flux
$$r$$: Axis coordinate
$R^{*}$: Radius of the circular surface
$$R_{d}$$: Radiation parameter
${\text{Re}}_{x}$: Local Reynolds number
$s$: Positive stretching constant
$$Sc$$: Schmidt number
$$S_{1} ,S_{2}$$: Chemical species
$T_{\infty }$: Convective temperature at infinity
$T_{f}$: Fluid thermal conductivity
$U$: Component of velocity
$u_{w}$: Stretching velocity along the *x-*direction
$v$: Component of velocity
$$x$$: Axis coordinates

## Greek symbols

$\alpha$: Dimensionless temperature difference
$$\alpha^{*}$$: Thermal diffusivity
$\delta$: The ratio of the diffusion coefficient
$\lambda {}_{1}$: Heat generation parameter
$\mu_{f}$: Fluid dynamic viscosity
$\mu_{nf}$: Nano-fluid dynamic viscosity
$\Phi$: Nanoparticles solid volume fraction
$$\psi$$: Dimensionless concentration
$\rho$: Density of solid nanoparticles
$\rho_{nf}$: Nano-fluid thermal conductivity
$\left( {\rho C_{p} } \right)_{f}$: Fluid heat capacity
$\left( {\rho C_{p} } \right)_{nf}$: Nanofluid heat capacity
$\rho_{f}$: Fluid thermal conductivity
$$\sigma$$: Electrical conductivity
$\sigma_{nf}$: Modified thermal diffusion
$\tau_{yx} ,\tau_{rx}$: Shear stress at the wall
$$\theta$$: Dimensionless temperature
$$\theta_{w}$$: Temperature difference
$\xi$: A scaled boundary layer coordinate
